# The use of telephone communication between nurse navigators and their patients

**DOI:** 10.1371/journal.pone.0227925

**Published:** 2020-01-24

**Authors:** Brody Heritage, Clare Harvey, Janie Brown, Desley Hegney, Eileen Willis, Adele Baldwin, David Heard, Sandy Mclellan, Virginia Clayton, Jamin Claes, Melanie Lang, Venessa Curnow

**Affiliations:** 1 College of Science, Health, Engineering and Education, Murdoch University, Perth, Western Australia; 2 School of Nursing, Midwifery and Social Science, Central Queensland University, Townsville, Queensland, Australia; 3 School of Nursing, Midwifery and Paramedicine, Curtin University, Western Australia, Australia; 4 Research Division, Central Queensland University, Brisbane, Australia; 5 Queensland, Australia, and School of Nursing, University of Adelaide, South Australia, Australia; 6 College of Nursing & Health Sciences, Flinders University, Adelaide, South Australia, Australia; 7 School of Nursing, Midwifery and Social Science, Central Queensland University, Mackay, Queensland, Australia; 8 Cairns Hospital and Health Service, Cairns, North Queensland, Australia; 9 Torres and Cape Hospital and Health Service, Cairns, North Queensland, Australia; University of Auckland, NEW ZEALAND

## Abstract

**Background:**

Hospitals and other health care providers frequently experience difficulties contacting patients and their carers who live remotely from the town where the health service is located. In 2016 Nurse Navigator positions were introduced into the health services by Queensland Health, to support and navigate the care of people with chronic and complex conditions. One hospital in Far North Queensland initiated an additional free telephone service to provide another means of communication for patients and carers with the NNs and for off-campus health professionals to obtain details about a patient utilising the service. Calls made between 7am and 10pm, seven days per week are answered by a nurse navigator.

**Aim:**

To report utilisation of the service by navigated clients and remotely located clinicians compared to use of navigators’ individual work numbers and direct health service numbers. We report the reason for calls to the free number and examine features of these calls.

**Methods:**

Statistical analysis examined the call reason, duration of calls, setting from where calls originated and stream of calls. Interactions between the reasons for calls and the features of calls, such as contact method, were examined.

**Results:**

The major reason for calls was clinical issues and the source of calls was primarily patients and carers. Clinical calls were longer in duration. Shorter calls were mainly non-clinical, made by a health professional. Setting for calls was not related to the reason. The most frequent number used was the individual mobile number of the NN, followed by the hospital landline. Although the free number was utilised by patients and carers, it was not the preferred option.

**Conclusion:**

As patients and carers preferred to access their NN directly than via the 1800 number, further research should explore options best suited to this group of patients outside normal business hours.

## Introduction

Globally, there is a significant increase in the prevalence of patients with chronic and complex conditions, brought about by various factors including improved health care, technology, improved life expectancy, and lifestyle factors [1]. Patients with chronic conditions require care over extended periods, which often spans multiple service providers and clinical specialities [2]. These interventions are not only costly but there is also clear evidence of fragmented care across the services, increased polypharmacy and patients becoming lost in the system, either because of miscommunication itself, or because patients do not understand what they have to do to manage their care [3]. Compounding these issues, are those related to poor health literacy, geographical isolation, and cultural disparity, in addition to known institutional discrimination for patients who are deemed non-compliant or frequent presenters to hospital [4, 5].

Health systems are looking for ways to address the complex needs of patients living with chronic conditions, with integrated care, improved self-management and hospital avoidance being high on the agenda [6, 7]. Although co-ordinated care has been identified as important, much of it is still provided in a system that caters for episodic, single condition, acute care, rather than a protracted care journey [8]. There is plethora of literature demonstrating that experienced nurses can effectively co-ordinate chronic and complex care, working with(in) an interdisciplinary team to ensure that care management is comprehensive [9, 10]. Further, in order to provide patients with culturally and clinically appropriate care, the needs of the patient should remain central to planning. The ‘best-fit’ care includes what Browne and colleagues (p. 5) call “inequity-responsive, contextually-tailored care” [11]. This approach encourages services to provide care based on levels of co-ordination, provided by professionals with different levels of clinical skill and scope of practice, matched to differing levels of patient need [12]. It also calls for a service that is accessible 24 hours a day, seven days a week. Co-ordinating chronic and complex care, where the patients know they have an accessible central contact, has been shown to reduce patient anxiety, thereby increasing a patient’s capability to manage their own care [13]. The rationale for this approach to service delivery is that patients and carers know who they can call for advice, and that they can call as soon as they feel the need to, in order to prevent a worsening of their condition. Coordinated care also fosters the patient’s ability to maintain autonomy in health care decisions [14]. Failure to support people living with long term conditions at home can translate to admissions to emergency departments that could be avoided with earlier intervention, advice or support [15].

In Far North Queensland additional impediments to effective navigation include geographical remoteness with 80% of the population living in a rural area geographically dislocated from the tertiary and specialist health services, and a further 8% being considered remotely located from the services [16]. There is also higher than state average social and economic disparity[17]. Moreover 60% of the population accessing health services identify as Aboriginal and Torres Strait Islander peoples, yet this population group represents just 14% of the total population in Far North Queensland. Cultural, social, economic and geographical disparity impede navigation services for people living away from the central health service located at Cairns, particularly those of First Nations background [18].

## Background

This paper analyses data collected on the use of a free-call telephone communication service between patients, their carers and healthcare professionals to nurse navigation services, the hub for which is located at the tertiary hospital in Cairns. Patients who are navigated are supported by a designated nurse navigator who maintains contact with the patients once they leave hospital. The analysis forms the first stage of a project that explores options for the delivery of an extended hours, seven-day a week nurse navigator service for patients and their carers living in Far North Queensland, who are Aboriginal and Torres Strait Islander, and living with chronic and complex conditions. In addition to the navigated patients and their carers, the telephone number, which is answered by a nurse navigator, was also provided to health professionals located across Far North Queensland.

Nurse navigators were initiated by Queensland Health in 2016 to assist patients with chronic conditions, with a focus on decreasing hospital avoidance, improving health literacy, and providing a conduit for patients being managed by an integrated care team [19, 20]. Nurses employed into these positions are experienced registered nurse clinicians with a broad understanding of the health system, who are able to guide and advise patients and their families on how to navigate their health journey across multiple services (government and non-government) that provide care to people with chronic conditions within acute and community settings. Nurse navigators have an extensive clinical background, with many holding a postgraduate qualification in their clinical area of practice. Across the state, nurse navigators are located in various departments and settings, for example respiratory, stroke, community or general acute medical settings. The navigator role description describes their work as 1) co-ordination of patients across a journey of care, working with a multidisciplinary team; 2) being the central point of care, engaging all stakeholders (community and acute service); 3) improving patient outcomes through evidence-based practice; and, 4) facilitating systems improvement as a clinical leader and change agent [21]. The nurse navigators identified in this paper are employed as systems navigators and are based in the tertiary health service (Cairns Hospital) which serves as the region’s specialist hub. Patients are frequently referred to the navigator service through an in-patient admission. Although some of the patients live within a 50km radius of Cairns, many live in regional and remote areas, linked into regionally located health services and clinics across the North Queensland region. All the patients who are referred to this navigator service have multiple chronic conditions, many of whom have more than three conditions simultaneously and therefore their care is complex, requiring multiple service and specialist input.

People with chronic and complex conditions require individualised care that is socially and culturally responsive, fostered through trusting relationships between the health professionals, families and the people receiving care and that promotes enhanced self-care [15]. Whilst Queensland Health navigator services have achieved gains in patient self-management and hospital avoidance, in Far North Queensland distance to tertiary care creates an additional barrier, with 88% of patients having to leave their community for specialist management of their chronic condition(s), particularly when a change of condition or exacerbation of an existing condition occurs. On return to their communities, often with a referral to local health care professionals (small rural hospital, family physician, remote area nurse), they may be lost to follow-up by the discharging, tertiary health service. Adding to the complexity in care provision, specialist and community care in rural communities is frequently out-sourced to non-government organisations (NGO), making it confusing for patients who do not know who to contact, or have to speak with staff who do not understand their medical history [22]. Nurse navigators have indicated that follow-up is problematic, particularly where there is no permanent telephone contact or fixed residential address. In an attempt to remediate this, Cairns and Hinterland Hospital and Health Service (Cairns HHS) trialled a 1800 number for patients to be able to contact a nurse navigator between 7am and 10pm, seven days per week. This was provided in addition to existing 24 hour health service contact, and the direct contact available to the patients with their designated navigator, available five days a week between 8am and 4pm.

The 1800 number is a free-to-caller service, answered by a navigator who may be any of the navigators in the health service, rostered to take calls. Information about the 1800 number was circulated to patients on admission to the navigator service, and to all Far North Queensland regional health services, and to any health professional working with patients with chronic and complex conditions. Refrigerator magnets and fliers were circulated widely in Far North Queensland to let people know about the 1800 number. Thus, the service was not limited to patients of the Cairns and Hinterland Hospital and Health Service, but also their families and health professionals who were managing one of the navigated patients in any area within Far North Queensland. The rationale for the inclusion of health professionals having access to the 1800 number focuses on the geographical dislocation between the hub of specialist services in Cairns, and those rural and remote locations distantly located, allowing for an accessible contact direct to the navigators. Being able to speak with a navigator located at Cairns Hospital facilitates direct navigated advice and streamlined referrals as required.

## The study

The focus of this research was to identify what call services were most often utilised by navigated patients and their carers. A secondary research question was related to which call service was most utilised by health professionals who worked externally to the tertiary referring hospital seeking more information related to navigated patients and /or the navigation service.

Calls from three telephone contact methods/numbers to Cairns Hospital, available to navigated patients, their carers and health professionals associated in the care, were monitored between November 19^th^ 2018 and January 30^th^ 2019, the data from which was collected for analysis. The three numbers monitored during this time were the usual contact made available to all people wishing to contact Cairns Hospital; individual work numbers carried by seven nurse navigators from 8am to 4pm, Monday to Friday; and the 1800 number dedicated to those patients and their carers being navigated, the location of the phone being with a rostered navigator 7am to 10pm, seven days per week.

### Aims

The aims of the study were to address the following questions regarding the characteristics of use for the navigator phone services:

1. What phone service was called (hospital or NN individual or 1800)
2. What were the most common reasons for calling the phone service?
3. Were specific features of the calls more common in comparison to others?
4. Was there an interaction between the call features and the reasons for the phone calls?

### Design

Data collection reflected a cross-section of logged phone calls between the dates of November 19^th^ 2018 and January 30^th^ 2019 to the service (hospital), to the 1800 number and as reported by seven navigators (individual work mobile number). The database that was used to collate call information existed prior to this research, therefore this study used existing records (Appendix 1—Supplementary data file). No patient records were used in this study. Only statistical information relating to calls to the 1800 number, nurse navigator work mobiles and the hospital main reception number were used. For the period of the data collection, the navigator service used a spreadsheet to document call reasons, call duration, call setting and call stream. Calls logged according to the following classifications were used in the data analyses:

- Call Reasons—classified as either clinical issues, practical issues related to patients coping at home, patient referrals, social issues, or were classified as an ‘other’ category if they did not fit in the former classifications.
- Call Duration—classified as being of a short (0–5 minutes), moderate (5–10 minutes), or longer (>10 minutes) duration.
- Call Setting—classified as being calls generated from either a community non-government organisation, inpatient service, the patient’s home, or a setting outside of the former categories (‘other’).
- Call Stream—classification included calls within the categories of patient or carer, medical, nursing, or a general ‘other’ classification for calls that did not fit within the former categories.

A Bayesian count model [23], using a Poisson distribution to model the positive count values of calls that fell under corresponding categories, was used. A Bayesian approach was employed in favour of the traditional null-hypothesis significance testing (NHST) approach (e.g., a *χ*^2^ for contingencies non-parametric analysis) due to the well-discussed difficulties with the core premises of NHST (for example, see Kruschke and Liddell, 2017), and the general ambiguity of a significant *χ*^2^ value in the context of the aforementioned analysis [23]. Consequently, parameter estimates of the main effects (i.e., Reason for Call categories and their proportionate frequencies), in addition to the interaction between effects were investigated per our research questions.

Due to the array of possible main effect contrasts to examine (e.g., Call Setting’s 4 levels have 10 potential main effects to examine), we opted to examine the most frequent category (e.g., home-based calls) versus each less-frequent category individually (e.g., home-based calls versus community non-government organisation / inpatient / OPD / other), and the average of the latter categories to establish the general odds of the most frequent category. Similarly, interactions presented the potential of a large array of interactions between the levels of call reason against call features for each model (i.e., the potential number of main effects of call reason multiplied by the potential number of main effects for call features). Due to the study’s primary focus on the reasons behind using the service, we therefore opted to compare each level of call reason (e.g., Clinical) versus the average of the remaining levels of call reason (e.g., non-Clinical), and the subsequent potential interaction with most-frequent level of a call feature (e.g., direct calls from a mobile phone) versus the average of the remaining levels of a call feature (e.g., calls not from a mobile phone). Estimates of the posterior parameters, in terms of their 95% Highest Density Interval (HDI) (i.e., the range of log-odds estimates when comparing the frequency of the categories of a call feature) against a Region of Practical Equivalence (ROPE; a range of the parameter estimates with no practical difference from an expected value, such as no difference between the category frequencies), was examined for each research question [23].

As the Poisson model parameter coefficients examined as part of the research questions were calculated as log-odds coefficients, exponentiating the log-odds coefficients to calculate odds-ratio coefficients was conducted for each comparison in order to facilitate clearer interpretation. Each model was tested using Kruschke’s syntax in *R* software, using *JAGS* [24]. Estimates of the model priors used Kruschke’s uninformative default priors in each instance of parameter testing. Well-mixed sampling chains of the JAGS models, effective sample sizes in excess of 10000 for the model parameters, and no evidence of autocorrelation were inspected prior to interpretation of each model.

### Ethics

Human research ethics approval for the project was provided under the multi-site ethical approval of the state-wide nursing navigator evaluation (Queensland Health HREC/18/QTDD/8).

## Analysis

In total, 280 calls were received between the data collection dates. Approximately 98% of these calls were received between Monday to Friday, and during regular working hours (7am to 4pm). On average, most calls (57.5%) were within 0–5 minutes in duration. Call reasons were most often clinical (37.1%) in nature, and calls reaching the service were most commonly sourced from nurse navigator mobile phones (47.5%) in comparison to other contact methods. The patient or carer stream (28.6%), or nursing stream (26.8%), were the most common call streams, and the patient’s home (28.9%) was the most common setting calls were placed from.

While the call classifications outlined in the prior research questions were sufficiently captured during this period (i.e., missing data in the variable crosstabulations examined was not present, and the consequential positive count of cases was suitable for the underlying Poisson distribution used in the later-outlined analysis), some call classifications had small marginal counts (i.e., <10 cases in total for a classification). An example of the latter was calls placed by Indigenous Hospital Liaison Officers to the navigator service, where three counts were recorded in total during the data collection period. Classification categories with small marginal counts consistent with the latter example were excluded from further analysis.

### Call reason

To examine whether calls being placed to the navigator service varied in frequency on the basis of the reason for the call, an examination of reasons for the call was conducted prior to examining the interactions with other variables. S1 Table outlines the log-odds coefficients, their respective odds-ratios, and the lower/upper HDI and proportion of overlap with the ROPE for the call reason levels. In terms of the main effects of call reason, clinically-based calls were the most common call reason, ranging between approximately 2.36 to 2.66 times more likely to be logged in the call register in comparison to practical, social, or other call reasons. In general, clinical calls were 2.54 times more likely to the reason for a call being logged by the service in comparison to all other potential call reasons. The general lack of an overlap between the ROPE and the lower and upper boundaries of the parameter HDI for the call reason comparisons, as detailed in S1 Table, further cemented the dominance of clinically-based calls being the primary reason for individuals to use the service.

### Call length

S1 Table outlines the main effects for call duration comparisons, where short calls (0–5 minutes in length) were approximately 2.43 times more likely to occur in comparison to moderate (6–10 minutes) calls. Furthermore, moderately long calls were 2.66 times more likely to be logged in the service records in comparison to longer (≥11 minutes) calls. When comparing short calls to the moderate or longer call lengths to the service, shorter calls were clearly more frequently reflected in the call logs of the service (approximately 3.89 times more likely than moderate or longer calls). Echoing the findings related to call reasons, the enhanced probability of shorter call lengths to the service did not suggest any overlap between the boundaries of the HDI and the ROPE for this model.

To examine the prospective dependency between the reason for a call being made its respective call length, interactions between the call reasons (i.e., clinical, practical, etc.) and call durations (short calls vs. moderate or longer calls) were examined. Some evidence of an increase in the odds of non-clinical calls being shorter in length (an approximate change in odds of 69%), or non-social calls similarly being more likely with shorter calls (approximately 96% more likely) was found. However, the non-marginal overlap between the HDI of these interaction terms and the ROPE (4.09% and 3.34% overlap per the aforementioned effects, see S1 Table) diminished our confidence in stating an interaction between call reason and duration for these effects. Alternatively, calls that did not fit any reason for call classification category (‘Other’ calls) were approximately 3.09 times more likely to be of a short duration compared to a moderate or longer duration. The latter effect was unlikely to be zero due to the marginal HDI and ROPE overlap for this term, therefore evidence of dependency between call reason and duration was noted for this effect. Alternatively, a call classified as having a practical call reason demonstrated no interaction with call duration as outlined in S1 Table. Consequently, while convincing evidence for ‘Other’ calls to the service to be of a short length was found, the remaining interactions demonstrated less-convincing odds of an interaction for the logged calls.

### Call setting

Users of the service were approximately 2.38 times more likely to be patients calling from their home in comparison to all other settings on average. Calls from patient homes were approximately 3.40 times more common in comparison to inpatient settings, and 3.57 times more common compared to OPD settings, on average. While the aforementioned comparisons demonstrated marginal overlaps between HDI boundaries and the ROPE as demonstrated in S2 Table, calls from patient homes were marginally (approximately 37%) more common in comparison to calls from community non-government organisations. The HDI boundary and ROPE overlap suggested that this difference was less-able to be interpreted as non-zero in nature, thereby making it difficult to rule out a practical equivalence in the frequency of community non-government organisation and home-based call settings recorded by the service. Consequently, the forthcoming interactions examined between call reason and call setting included an additional set of interactions that examined patterns related to home setting interactions, and community non-government organisation setting calls (S2 Table).

To examine whether the classification categories of call reason were dependent on the call setting classification, we examined whether reason (i.e., clinical, social, etc.) demonstrated a non-zero interaction with call settings (i.e., the home setting or community non-government organisation versus other settings calls were received from). As demonstrated in S2 Table, no compelling evidence for higher odds of the call being of a clinical, social, or practical nature when the call was received from the patient’s home versus other potential settings was found. Some evidence of a potential interaction between the ‘Other’ call reason and whether the call setting was the patient’s home or elsewhere was noticed, with an approximate 99% increase in the odds of the call setting being the patient’s home if an ‘Other’ call was received. However, the small overlap between the HDI and ROPE of this interaction term (approximately 2.59%) suggested some uncertainty as to whether this interaction could be convincingly interpreted. A similar set of findings were produced when examining the interactions between community non-government organisation setting calls versus other call settings, and whether there was a relationship to certain call reasons. All HDI and ROPE overlaps for the latter interactions were notable (see S2 Table) and were therefore unable to be interpreted with any clarity. In summary, limited evidence of a dependency between the reasons for calls and the setting the calls were received from within the call logs was found, suggesting that these call reason and setting are likely to be independent of each other.

### Call stream

The stream of the calls logged by the phone service suggested some variation in the odds of a particular stream using the service. Patients and their carers were generally the most common stream of service users, being approximately 2.23 times more likely to use the phone service in comparison to any other stream recorded in the data (see S3 Table). Patients and their carers were approximately 5.01 times more likely to use the service in comparison to calls received from a general medical stream. The two effects demonstrated no overlap between the HDI and ROPE, allowing confidence in the interpretation of an enhanced likelihood of patients and their carers using the service in these instances. In comparison to users of the service from a nursing stream however, no notable change in the odds of a call being logged from the patient/carer stream or the nursing stream was observed on the basis of the overlap (approximately 21.86%) between the HDI and ROPE for this effect. Due to ambiguity in the degree of differentiation in calls from the patient or their carer to the service, or its use by other nurses, both call stream categories were examined in the forthcoming interaction terms. Calls from the patient/carer stream were approximately 1.50 times more likely in comparison to calls from the ‘Other’ stream of calls not captured by the aforementioned categories. Due to the minor overlap in the HDI and ROPE of this effect though (approximately 3.39%), it was unclear whether this effect was interpretable.

Regarding the interactions between call reason and call stream, no evidence of dependency between the call factors was interpretable for clinical, social, or practical calls. A potential dependency between the likelihood of calls *not* categorised as having an ‘Other’ reason, and the likelihood of the calls stemming from the patient/carer stream, was noted. This combination of call factors was approximately 86% more likely to be observed in comparison to patient/carer stream calls being categorised as having an ‘Other’ reason for the call, however the small overlap between the HDI and ROPE of this interaction term (approximately 2.83%) made it less clear as to whether this was an interpretable effect. A similar finding for the nursing stream using the service and an enhanced (79% more likely) odds of the reason for the call being clinical in nature was noted, however the small overlap between the HDI and ROPE of this effect (3.67%) did not allow confidence in interpreting this finding. In summary, limited evidence of a dependency between call stream factors and the reason for the calls being made was apparent in the call log data.

### Contact method

When examining contact methods with the nurse navigators (i.e., directly via mobile phone, directly via hospital landline, or via the service’s 1800 number), direct calls via mobile phone was the most common method of contact with the NN service, being approximately 2.46 times more likely than the alternative contact methods. Mobile phone calls versus the calls from a hospital landline to the NN service heavily favoured the mobile phone contact method, as it was approximately 4.06 times more likely to be the recorded method of contact (see S4 Table). Additionally, a direct call to the service via mobile phone was approximately 49.2% more likely in comparison to the use of the 1800 number to reach the service. A minor overlap between the HDI and ROPE was noted for the latter (approximately 1.87%), therefore some ambiguity about the interpretability of this effect remained. We therefore opted to conduct a second series of interaction analyses comparing the call reasons associated with the 1800 number use vs. mobile/landline use as outlined in the forthcoming section.

The interactions between contact method and the reason for the call being placed to the service suggested no strong evidence of dependency between these factors. Calls *not* classified as an ‘Other’ reason for calling were approximately 82.7% more likely to arrive at the NN service via a direct call from a mobile phone, however the overlap (approximately 6.32%) between the HDI and ROPE of this interaction suggested uncertainty about the interpretability of this interaction effect. The remaining interaction effects, as outlined in S4 Table, presented greater overlap between their HDI and ROPE estimates, and were therefore not interpreted. We therefore concluded no notable dependency between the method of contact and the reason for calling the service was present in the data.

## Discussion

When considering the methods in which people used the phone service, patients and their carers preferred to contact their designated navigator via the service in comparison to other potential call streams. Additionally, we found a degree of practical equivalence in service use between patients/carers and nurses (i.e., nurses calling the nurse navigators via the service). Direct contact with the nurse navigator via mobile phone during regular working days and standard office hours was the most commonly used form of contact with the nurse navigator service. The latter finding suggests that the patient preference is to call their designated nurse navigator directly when requiring support. Limited evidence of call features (e.g., duration) being contingent on the reason for the call being placed to the nurse navigator service was found, outside of an increased prospect of ‘Other’ calls reaching the service being short in duration (0 to 5 minutes). The reasons for calls to the 1800 number were mainly of a clinical nature, and more often generated from a carer or a patient. These calls were likely to be sourced from the patient or carer’s own home, or a community non-government organisation. These findings addressed our research questions regarding the most common call reasons, call features, and the (limited) interactions between these call aspects.

The use of technology to reach patients has been explored in other countries, with the use of video conferencing, telephone consultations, nurse-led telephone triage and texting using mobile phones proven to be effective [25, 26]. The Cairns Hospital 1800 number was established so that patients would have extended access to health care advice from a nurse navigator. It appears this is not a preferred option for the most frequent users of the service (patients, their carers, and nurses), who prefer calling the navigator directly via their mobile phone, during regular working days and hours of operation (98% of the calls for the latter). While miscommunication between services about a patient’s care arrangements, and between the patient-health practitioner interaction are known to potentiate fragmented care [27], the uptake of the direct calls to the nurse navigators may suggest greater accessibility in communication between the nurse navigators, their patients, and their colleagues. However, the preference for directly calling the nurse navigator did seem to suggest a lesser degree of uptake for the 1800 number service itself, and marginal contact with the nurse navigator outside of regular working days and operating hours. Consequently there remains the challenge of how best to provide patients and their carers with a means of contact outside business hours, especially as people living with multiple chronic conditions require a more individualised approach to care over extended periods of time, and a central point of contact in order to reduce the fragmentation that exists in multi-service care provision [27].

Internationally, the use of telehealth services is encouraged and has been shown to be a successful way of reaching people living with long term conditions [28]. Queensland Health has adopted this approach in an effort to reach more people in the regional areas [29]. The Primary Health Network identifies that telehealth is well used by clinicians in the main regional centres but is silent on patient capacity to reach such services, or its use outside of the main service hubs [16]. Further research is required to find out what is the best way for people living with long term conditions in FNQ to access services via telephone, especially outside “business” hours.

Encouragingly, a complementary interpretation to our finding of the popularity of direct calls to the nurse navigator via mobile is that patients and their carers felt sufficiently comfortable in their interaction with the nurse navigator service provider, and therefore felt as though they can (or should) call their nurse navigator directly, without needing to interact via the mediatory service of the 1800 number. This may suggest the importance of practitioner-patient accessibility, or relationship quality, when considering service provision to remote patients, however the latter interpretation requires further substantiation in future research.

## Limitations

This study does not report on phone usage against emergency department occasions of service, nor does it explore patient or nurse views of why they prefer one contact option over others. These two factors will be explored in the next stage of this project so that a comprehensive needs analysis from a community perspective can be examined in more detail. Additionally, the cost of maintaining a navigator presence for extended hours was not explored in this first stage of the project, although the nurse navigators did indicate that their work continued as usual after hours, so that their time remained productive.

Furthermore, due to the free entry of caller names (instead of the use of unique caller identifiers) within the dataset used for analysis, which was unfortunately inconsistent with the use of pre-established categorisation options for the other variables, ambiguity in relation to caller identities existed. The caller name information that was collected was therefore not included for analysis. While we recognise the limitation in treating the calls as stemming from unique callers during analysis, the alternative option of guessing unique caller identities when facing incomplete information introduces a potential new limitation. Future data collection should collect unique anonymous caller ID information as a potential remedy to this limitation for reliable inference. Additionally, given the coding of patient or caregiver sourced streams for calls as a combined category, this coding level allowed less granularity in determining whether patients or carers equivalently used the service.

## Conclusion

Supporting people and their carers living with long term conditions is complex and expensive, with integrated care and hospital avoidance being the focus of attention. International literature suggests various models of care to assist the patient/carer on that journey, many of which include the use of virtual technology for health services and patients to remain connected. However, in this study, while there has been some uptake from remotely located health care professionals to obtain patients who are in the NN service, do make calls to the service, mostly from their homes and for a clinical inquiry, they are still calling their allocated NN on the NN mobile phone number. Despite the rhetoric of technological enhanced care, there are practical, cultural, and geographical considerations that need to be addressed. It is this perspective that the project will explore in the next stage of this research.

## Supporting information

S1 Table Main and interaction effects for frequencies of call reason and call duration categories. (DOCX)

S2 Table Main and interaction effects for frequencies of call reason and call setting categories. (DOCX)

S3 Table Main and interaction effects for frequencies of call reason and call stream categories. (DOCX)

S4 Table Main and interaction effects for frequencies of call reason and contact method categories. (DOCX)

S1 File Supplementary data file. (DOCX)
