# Supplementary material for: The use of telephone communication between nurse navigators and their patients
Source: PLoS One. 2020 Jan 24;15(1):e0227925. doi: 10.1371/journal.pone.0227925 (PMC6980411; doi:10.1371/journal.pone.0227925)
Supplement: S1 Table — (DOCX) [file pone.0227925.s001.docx]

**S1 Table 1 Main and interaction effects for frequencies of call reason and call duration categories**

| Variable Contrast | Log-Odds ^a^ (95% LHDI,UHDI) | Odds-Ratio (95%LHDI,UHDI) | ROPE ^b^ Overlap % |
| --- | --- | --- | --- |
| *Call Reason* |  |  |  |
| Clinical vs. Other | 0.858 (0.383,1.331) | 2.358 (1.467,3.786) | 0.020% |
| Clinical vs. Practical | 0.977 (0.547,1.451) | 2.655 (1.728,4.269) | 0.000% |
| Clinical vs. Social | 0.934 (0.514,1.368) | 2.544 (1.672,3.927) | 0.013% |
| Clinical vs. Else ^c^ | 0.931 (0.608,1.267) | 2.537 (1.836,3.551) | 0.000% |
| *Call Duration* |  |  |  |
| 0-5 vs. 6-10 | 0.887 (0.575,1.232) | 2.427 (1.778,3.427) | 0.000% |
| 6-10 vs. 11-20 | 0.979 (0.481,1.511) | 2.663 (1.618,4.531) | 0.000% |
| 0-5 vs. Else | 1.359 (1.082,1.713) | 3.893 (2.949,5.546) | 0.000% |
| *Call Reason*Duration* |  |  |  |
| (Else vs. Clinical)*(0-5 vs. Else) | 0.527 (0.015,1.114) | 1.694 (1.015,3.046) | 4.093% |
| (Practical vs. Else)*(0-5 vs. Else) | 0.047 (-0.649,0.726) | 1.048 (0.522,2.067) | 22.340% |
| (Else vs. Social)*(0-5 vs. Else) | 0.675 (-0.007,1.416) | 1.963 (0.993,4.119) | 3.340% |
| (Other vs. Else)*(0-5 vs. Else) | 1.128 (0.523,1.989) | 3.091 (1.687,7.305) | 0.020% |

*Note.* Per Kruschke (2018), the ROPE employed is between -0.10 and 0.10 to reflect approximately a 10% change in the odds-ratio. ^a^ The mode of the log-odds coefficient estimate, and lower and upper boundaries of the 95% Highest Density Interval. ^b^ Region of Practical Equivalence. ^c^ ‘Else’ refers to all other categories than the other listed category for a variable (e.g., Clinical calls compared to non-Clinical calls). * Interaction between the main effects presented.
