# Supplementary material for: The use of telephone communication between nurse navigators and their patients
Source: PLoS One. 2020 Jan 24;15(1):e0227925. doi: 10.1371/journal.pone.0227925 (PMC6980411; doi:10.1371/journal.pone.0227925)
Supplement: S2 Table — (DOCX) [file pone.0227925.s002.docx]

**S2 Table 2 Main and interaction effects for frequencies of call reason and call setting categories**

| Variable Contrast | Log-Odds ^a^ (95% LHDI,UHDI) | Odds-Ratio (95%LHDI,UHDI) | ROPE ^b^ Overlap % |
| --- | --- | --- | --- |
| *Call Setting* |  |  |  |
| Home vs. Comm NGO ^c^ | 0.311 (-0.052,0.673) | 1.365 (0.949,1.96) | 11.027% |
| Home vs. Inpatient | 1.223 (0.721,1.724) | 3.396 (2.056,5.605) | 0.000% |
| Home vs. OPD | 1.273 (0.78,1.826) | 3.573 (2.181,6.21) | 0.000% |
| Home vs. Other | 0.594 (0.176,1.04) | 1.811 (1.192,2.83) | 0.573% |
| Home vs. Else ^d^ | 0.865 (0.541,1.178) | 2.375 (1.718,3.249) | 0.000% |
| *Interaction term* |  |  |  |
| (Clinical vs. Else)*(Home vs. Else) | 0.182 (-0.332,0.691) | 1.200 (0.718,1.996) | 24.900% |
| (Social vs. Else)*(Home vs. Else) | 0.248 (-0.343,1.009) | 1.281 (0.710,2.742) | 16.707% |
| (Else vs. Other)*(Home vs. Else) | 0.688 (0.040,1.401) | 1.990 (1.041,4.059) | 2.587% |
| (Practical vs. Else)*(Home vs. Else) | 0.194 (-0.369,0.930) | 1.214 (0.692,2.535) | 18.673% |
| (Else vs. Clinical)*(Comm NGO vs. Else) | 0.329 (-0.209,1.006) | 1.390 (0.811,2.736) | 13.113% |
| (Social vs. Else)*(Comm NGO vs. Else) | 0.346 (-0.190,1.257) | 1.414 (0.827,3.513) | 10.113% |
| (Else vs. Other)*(Comm NGO vs. Else) | 0.388 (-0.224,1.111) | 1.474 (0.800,3.038) | 11.593% |
| (Practical vs. Else)*(Comm NGO vs. Else) | 0.322 (-0.316,1.054) | 1.380 (0.729,2.870) | 14.980% |

*Note.* Per Kruschke (2018), the ROPE employed is between -0.10 and 0.10 to reflect approximately a 10% change in the odds-ratio. ^a^ The mode of the log-odds coefficient estimate, and lower and upper boundaries of the 95% Highest Density Interval. ^b^ Region of Practical Equivalence. ^c^ Community Non-Government Organisation. ^d^ ‘Else’ refers to all other categories than the other listed category for a variable (e.g., Clinical calls compared to non-Clinical calls). * Interaction between the main effects presented.
