# Supplementary material for: The use of telephone communication between nurse navigators and their patients
Source: PLoS One. 2020 Jan 24;15(1):e0227925. doi: 10.1371/journal.pone.0227925 (PMC6980411; doi:10.1371/journal.pone.0227925)
Supplement: S3 Table — (DOCX) [file pone.0227925.s003.docx]

**S3 Table 3. Main and interaction effects for frequencies of call reason and call stream categories**

| Variable Contrast | Log-Odds ^a^ (95% LHDI,UHDI) | Odds-Ratio (95%LHDI,UHDI) | ROPE ^b^ Overlap % |
| --- | --- | --- | --- |
| *Call Stream* |  |  |  |
| Patient/Carer vs. Medical | 1.612 (1.079,2.306) | 5.014 (2.941,10.033) | 0.000% |
| Patient/Carer vs. Nursing | 0.212 (-0.151,0.583) | 1.236 (0.86,1.791) | 21.860% |
| Patient/Carer vs. Other | 0.407 (0.066,0.833) | 1.502 (1.068,2.299) | 3.393% |
| Patient/Carer vs. Else ^c^ | 0.801 (0.437,1.116) | 2.227 (1.548,3.052) | 0.000% |
| *Interaction term* |  |  |  |
| (Else vs. Clinical)*(Patient/Carer vs. Else) | 0.002 (-0.500,0.595) | 1.002 (0.607,1.813) | 28.493% |
| (Social vs. Else)*(Patient/Carer vs. Else) | 0.246 (-0.382,0.993) | 1.279 (0.683,2.699) | 16.760% |
| (Practical vs. Else)*(Patient/Carer vs. Else) | 0.389 (-0.213,1.198) | 1.476 (0.808,3.313) | 9.940% |
| (Else vs. Other)*(Patient/Carer vs. Else) | 0.622 (0.037,1.449) | 1.863 (1.037,4.260) | 2.833% |
| (Clinical vs. Else)*(Nursing vs. Else) | 0.583 (0.019,1.194) | 1.791 (1.019,3.299) | 3.673% |
| (Else vs. Social)*( Nursing vs. Else) | 0.386 (-0.271,1.279) | 1.471 (0.763,3.593) | 10.740% |
| (Else vs. Practical)*( Nursing vs. Else) | 0.043 (-0.618,0.843) | 1.044 (0.539,2.323) | 22.047% |
| (Else vs. Other)*( Nursing vs. Else) | 0.008 (-0.591,0.642) | 1.008 (0.554,1.900) | 26.707% |

*Note.* Per Kruschke (2018), the ROPE employed is between -0.10 and 0.10 to reflect approximately a 10% change in the odds-ratio. ^a^ The mode of the log-odds coefficient estimate, and lower and upper boundaries of the 95% Highest Density Interval. ^b^ Region of Practical Equivalence. ^c^ ‘Else’ refers to all other categories than the other listed category for a variable (e.g., Clinical calls compared to non-Clinical calls). * Interaction between the main effects presented.
