# Supplementary material for: The use of telephone communication between nurse navigators and their patients
Source: PLoS One. 2020 Jan 24;15(1):e0227925. doi: 10.1371/journal.pone.0227925 (PMC6980411; doi:10.1371/journal.pone.0227925)
Supplement: S4 Table — (DOCX) [file pone.0227925.s004.docx]

**S4 Table 4. Main and interaction effects for frequencies of call reason and contact method categories**

| Variable Contrast | Log-Odds ^a^ (95% LHDI,UHDI) | Odds-Ratio (95%LHDI,UHDI) | ROPE ^b^ Overlap % |
| --- | --- | --- | --- |
| *Contact Method* |  |  |  |
| Mobile vs. Land ^c^ | 1.402 (0.991,1.880) | 4.063 (2.694,6.556) | 0.000% |
| Mobile vs. 1800 | 0.400 (0.124,0.721) | 1.492 (1.132,2.056) | 1.867% |
| Mobile vs. Else ^d^ | 0.901 (0.597,1.208) | 2.461 (1.817,3.348) | 0.000% |
| *Interaction term* |  |  |  |
| (Clinical vs. Else)*(Mobile vs. Else) | 0.001 (-0.536,0.45) | 1.001 (0.585,1.568) | 34.607% |
| (Social vs. Else)*( Mobile vs. Else) | 0.018 (-0.58,0.649) | 1.019 (0.560,1.914) | 28.840% |
| (Practical vs. Else)*( Mobile vs. Else) | 0.404 (-0.125,1.382) | 1.497 (0.883,3.984) | 9.840% |
| (Else vs. Other)*( Mobile vs. Else) | 0.603 (-0.023,1.291) | 1.827 (0.978,3.635) | 6.320% |
| (Else vs. Clinical)*(1800 vs. Else) | 0.001 (-0.460,0.553) | 1.001 (0.631,1.739) | 33.240% |
| (Else vs. Social)*(1800 vs. Else) | 0.064 (-0.464,0.865) | 1.066 (0.629,2.375) | 24.973% |
| (Else vs. Practical)*(1800 vs. Else) | 0.005 (-0.861,0.575) | 1.005 (0.423,1.777) | 26.313% |
| (Other vs. Else)*(1800 vs. Else) | 0.072 (-0.443,0.628) | 1.074 (0.642,1.874) | 29.240% |

*Note.* Per Kruschke (2018), the ROPE employed is between -0.10 and 0.10 to reflect approximately a 10% change in the odds-ratio. ^a^ The mode of the log-odds coefficient estimate, and lower and upper boundaries of the 95% Highest Density Interval. ^b^ Region of Practical Equivalence. ^c^ General hospital landline phone call. ^d^ ‘Else’ refers to all other categories than the other listed category for a variable (e.g., Clinical calls compared to non-Clinical calls). * Interaction between the main effects presented.
