# Supplementary material for: The use of telephone communication between nurse navigators and their patients
Source: PLoS One. 2020 Jan 24;15(1):e0227925. doi: 10.1371/journal.pone.0227925 (PMC6980411; doi:10.1371/journal.pone.0227925)
Supplement: S1 File — (DOCX) [file pone.0227925.s005.docx]

**S1 File – Supplementary Data File**

**Data Availability Statement:** The human research ethical approval for this study did not permit individual-level data to be made publicly available due to participant confidentiality and privacy requirements. Congruent with the study's human research ethics approval, all aggregate-level data is made fully available in this article's Tables and Supporting Information. The release of de-identified individual-level data would require ethical approval, therefore data requests by qualified and interested researchers may be sent to the Human Research Ethics office, Central Queensland University, email: [ethics@cqu.edu.au](mailto:ethics@cqu.edu.au)

| Rreason For Call | Duration Of Call | Count |
| --- | --- | --- |
| Clin | 00_05 | 52 |
| Clin | 06_10 | 39 |
| Clin | 11_20 | 11 |
| othCom | 00_05 | 57 |
| othCom | 06_10 | 7 |
| othCom | 11_20 | 3 |
| Pract | 00_05 | 26 |
| Pract | 06_10 | 12 |
| Pract | 11_20 | 3 |
| Social | 00_05 | 18 |
| Social | 06_10 | 11 |
| Social | 11_20 | 7 |
| #################################### | | |
| Reason | Fsetting | Count |
| Clin | ComNGO | 17 |
| Clin | Inpatient | 9 |
| Clin | OPD | 10 |
| Clin | Other | 18 |
| Clin | PatHome | 35 |
| othCom | ComNGO | 11 |
| othCom | Inpatient | 7 |
| othCom | OPD | 7 |
| othCom | Other | 23 |
| othCom | PatHome | 12 |
| Pract | ComNGO | 12 |
| Pract | Inpatient | 4 |
| Pract | OPD | 3 |
| Pract | Other | 2 |
| Pract | PatHome | 15 |
| Social | ComNGO | 12 |
| Social | Inpatient | 2 |
| Social | OPD | 1 |
| Social | Other | 4 |
| Social | PatHome | 14 |
| #################################### | | |
| Reason | Fstream | Count |
| Clin | Medcl | 7 |
| Clin | Nursing | 38 |
| Clin | Other | 12 |
| Clin | PtnCarer | 31 |
| othCom | Medcl | 6 |
| othCom | Nursing | 18 |
| othCom | Other | 20 |
| othCom | PtnCarer | 13 |
| Pract | Medcl | 1 |
| Pract | Nursing | 8 |
| Pract | Other | 6 |
| Pract | PtnCarer | 16 |
| Social | Medcl | 1 |
| Social | Nursing | 6 |
| Social | Other | 11 |
| Social | PtnCarer | 15 |
| #################################### | | |
| Reason | Contact | Count |
| Clin | 1800 | 35 |
| Clin | HospLanL | 14 |
| Clin | NavMob | 54 |
| othCom | 1800 | 27 |
| othCom | HospLanL | 14 |
| othCom | NavMob | 25 |
| Pract | 1800 | 13 |
| Pract | HospLanL | 1 |
| Pract | NavMob | 27 |
| Social | 1800 | 11 |
| Social | HospLanL | 5 |
| Social | NavMob | 20 |
| #################################### | | |
|  |  |  |
